# On the Application of Nitrate of Silver in Acute Tonsillitis

**Published:** 1853-09

**Authors:** 


*On the Application of Nitrate of Silver in Acute Tonsillitis.* By M. Herpin. M. Herpin states that he finds the application of nitrate of silver in substance to be a most excellent mode of abridging the duration of acute tonsillitis, preventing suppuration in persons liable to this occurrence. Even in the most intense cases, accompanied by great febrile action, he has not had to make more than three applications. If suppuration has already occurred, the application is of less avail, and is then, on account of the closure of the jaws, often impracticable. The application must be carefully and methodically made opposite a window. If the velum is inflamed it should be touched in passing from one tonsil to another, as also may the uvula—but as a spasm of the fauces is often then induced, this should be left to the last. If the application is made within the first twenty-four hours, a single one often suffices: and this happens in persons who are liable to relapse of this affection, and have already derived benefit from the caustic. If seen later, two applications at the interval of a day, or even three, are required, although the first at once checks the progress of the disease. More than twenty-four hours should never be allowed to elapse between the applications. Since he first recommended this practice, many of M. Herpin's colleagues at Geneva have adopted it, and with the best effects, in securing the rapid dispersion of a disagreeable though not a dangerous disease.—*L'Union Médicale* and *Medico-Chirurgical Review.*

